# Feeding Pasteurized Waste Milk to Preweaned Dairy Calves Changes Fecal and Upper Respiratory Tract Microbiota

**DOI:** 10.3389/fvets.2019.00159

**Published:** 2019-06-06

**Authors:** Georgina Maynou, Hugh Chester-Jones, Alex Bach, Marta Terré

**Affiliations:** ^1^Department of Ruminant Production, Institute of Agrifood Research and Technology (IRTA), Caldes de Montbui, Spain; ^2^Department of Animal Science, Southern Research and Outreach Center (SROC), Waseca, MN, United States; ^3^Catalan Institution for Research and Advanced Studies (ICREA), Barcelona, Spain

**Keywords:** calves, fecal microbiota, upper respiratory tract microbiota, waste milk, 16S rRNA gene sequencing

## Abstract

In the present study bacterial communities from both, the gastrointestinal and respiratory tract of pre-weaned dairy calves fed two different milk-feeding programs were characterized using 16S rRNA gene sequencing. Twenty female Holstein calves (38.8 ± 1.40 kg of BW) were fed pasteurized waste milk (pWM) containing residues of various antimicrobials. Twenty additional calves (38.1 ± 1.19 kg of BW) were fed milk replacer (MR) with similar nutrient composition (27.5% crude protein, 32.1% fat) compared to waste milk (28.6% crude protein, 30.0% fat) from day 1 to weaning at day 49 of study. Fecal samples and nasal swabs were collected on day 42 only from calves that were not treated with therapeutic antibiotics throughout the study, which were 8 MR and 10 pWM calves. To assess the impact of the two feeding regimes on the fecal and nasal microbiota, α and β-diversity measures were calculated, and the relative abundance of operational taxonomic units (OTUs) at different taxonomic levels was determined for each sample. In general, Chao1, PD Whole Tree, and Shannon diversity indices were similar for the fecal and nasal bacterial communities of calves regardless of the feeding regime. However, principal coordinate analysis based on unweighted Unifrac distances indicated differences in the structure of bacterial communities of calves fed milk replacer compared with those from calves fed pasteurized waste milk. The relative abundance of the *Streptococcaceae* family and the genus *Histophilus* was greater (*P* < 0.05) in the nasal microbiota of calves fed milk replacer than in those fed pasteurized waste milk. However, the genus *Prevotella* tended (*P* = 0.06) to be more relatively abundant in the respiratory tract of calves fed pasteurized waste milk than in those fed milk replacer. Differences in relative abundances of bacterial taxa in gut microbiota were only observed at the phylum level, suggesting that antimicrobial residues present in waste milk have a non-specific influence at a lower taxonomical level.

## Introduction

Growth and development of dairy calves are highly influenced by the composition and activity of their associated microbiota (1, 2). A classic example of the importance of the bovine microbiota is the rumen, where fermentation of dietary substrates by the microbiota results in the formation of short-chain fatty acids. Short-chain fatty acids are a major energy source for the host animal, and an important substrate for the development of the rumen epithelium (3, 4).However, in newborn calves, milk is primarily digested in the small intestine (5), and microbes colonizing the small intestine can contribute to intestinal homeostasis (6, 7), the stimulation of the immune system, and enhance the development of the intestinal epithelium (1, 7).

The type of feed offered to calves impacts the structure of the gut microbiota by providing different dietary substrates to bacterial communities (8–10). However, most of the studies evaluating the effects of different dietary regimes on the gut microbiota of calves have focused on the impact of solid feed (11–13), whereas little information is available about how different milk feeding regimes affect the microbial composition of the gut (5, 14, 15).

In dairy operations, different types of feed can be used during the preweaning period: milk replacer (MR), saleable whole milk, and non-saleable milk (i.e., containing traces of antimicrobials, colostrum, transition milk), also called waste milk (WM) (16). In spite of MR being the most common nutrient source used in calf feeding programs (17), the use of WM has gained popularity in recent years. This is due to the large volumes of WM associated with the increasing herd size of commercial dairies (14), and the cost advantage of WM over whole milk and MR (16, 18). Moreover, increased usage of on-farm pasteurizers has promoted this trend as the inactivation of bacteria through pasteurization greatly reduced the risk of transmitting infectious diseases through feeding WM. In fact, Godden et al. (19) demonstrated reduced morbidity and mortality as well as improved growth rates in calves fed pasteurized waste milk (pWM) compared to those fed conventional MR.

Additional concerns related to the use of WM arise from the presence of drug residues at sub-therapeutic concentrations which could increase the selection for resistant bacteria (20). Previous studies have reported increases in the prevalence of antimicrobial resistant bacteria in the gut and also respiratory microbiota of calves fed WM or pWM compared with those fed MR (21, 22). The effects of antimicrobials in animal feed on the gut microbiota have previously been evaluated in the gastrointestinal tract of monogastrics (poultry and swine) (23–25) and changes in the taxonomic composition of the gut microbiota as well as on the expression of microbial functional genes have been reported. However, there is little information concerning the effects of pWM containing antimicrobials on the respiratory tract of farm animals. Thus, the aim of the present study was to characterize the gastrointestinal and respiratory tract microbiota of preweaned calves fed either, standard MR or pWM containing antimicrobials residues using next generation sequencing technology.

## Materials and Methods

### Calf Management

The current study was conducted at the Southern Research and Outreach Center (SROC) at the University of Minnesota from July to November 2014 and was approved by the Animal Care and Use Committee (IACUC) of the same university under protocol number 1407-31648A. The SROC Calf and Heifer Facility raised Holstein dairy heifer calves coming from three commercial dairy operations in Minnesota. Calves were collected twice per week from the respective dairies at 2 to 5 days of age, and raised until 6 months of age. At the farm of origin, calves were offered at least 3 colostrum feedings of 3 to 4 L each within the first 24 h after birth and thereafter 2 L of mixed transition milk twice daily until their transportation to SROC. From arrival to 56 days of age, calves were housed in individual pens (2.3 × 1.2 m) in naturally ventilated calf barns divided into 2 rooms (~40 calves per room). Each individual pen was separated by panels to avoid direct contact between adjacent calves. The pens were bedded with clean shavings. After arrival at SROC and at day 56, calves were vaccinated against infectious bovine rhinotracheitis virus, parainfluenza-3 virus, and bovine respiratory syncytial virus (Inforce 3, Zoetis, Florham Park, New Jersey).

When symptoms of disease were observed, calves were treated against diarrhea with a combination of oral 800 mg sulfamethoxazole and 160 mg trimethoprim tablets (Bactrim, Amneal Pharmaceuticals, Bridgewater, New Jersey) at 1 tablet/40 kg BW twice daily during 1–3 days, and with an injectable solution of enrofloxacin (Baytril, Bayer, Shawnee Mission, Kansas, MO) at 7.5 to 12.5 mg/kg BW/day during 1–2 days to treat against respiratory diseases. In cases of bloat, 5 mL of procaine G penicillin were administered i.m and an additional 15 ml per calf were administered orally (PenOnePro, Labs LTD, Newry, Northern Ireland).

### Experimental Treatments and Sample Collection

A subset of the calves used in this experiment were part of a study comparing three different milk replacers and pWM (26). The companion study did not interfere with our experiment. A total of forty female Holstein calves were enrolled in the present study (3.5 ± 1.15 d of age and 39.3 ± 4.25 kg of BW) and were assigned to 1 of 2 milk feeding treatments by farm of origin and BW: (1) a non-medicated all-milk protein MR (26% crude protein and 31% fat on a dry matter basis; Milk products Inc., Chilton, Wisconsin) diluted to 12.5% dry matter or (2) pWM (28.4% crude protein and 30.1% fat on a dry matter basis) containing traces of antimicrobials as analyzed. Starting on the day of arrival, calves in both treatment groups were fed 0.34 kg of dry matter (either from MR or pWM) per feeding twice daily for 42 days. The waste milk used to feed calves was collected from one single local dairy 2 to 3 times weekly throughout the study and stored at 4°C in a milk tank until pasteurization at 63°C for 35 min. For each milk load, the milk solids content of pWM was determined using a brix refractometer (Spartan Refractometer, Model A 300 CL, Spartan, Tokyo, Japan) to equalize nutrient intakes between the two treatments. For each WM load, a milk sample before pasteurization was analyzed for fat, lactose, protein, total non-fat solids by infrared spectroscopy, and somatic cells by a cell counter (Minnesota DHIA Laboratory, Zumbrota, MN). The total bacteria and coliforms in both WM and pWM were enumerated on PCA and McConkey agar, respectively, for each milk load at the University of Minnesota Veterinary Diagnostic Laboratory (St. Paul, Minnesota) to confirm that the pasteurization treatment decreased total bacterial and coliform counts by 3-log. For this analysis, one WM sample was collected before pasteurization and two samples after pasteurization. Although concentration and composition of the antimicrobial residue in WM might have varied depending on the number of treated cows on the farm and the type of treatments that were administered, each batch of WM was screened for β-lactam residues after being pasteurized using a commercial enzyme-linked receptor-binding assay test (SNAP β-lactam test) (Idexx Laboratories Inc., Westbrook, Maine). Most of the antimicrobials used to treat cows on the farm the WM originated from belonged to the β-lactam family (mainly cephalosporins rather than penicillin), and β-lactam residues were detected in each load of WM throughout the study. Other antimicrobials, such as lincosamides, may have also been present in the WM because they were also used on the farm that provided the WM.

From day 1 to 42 of the study, calves had free access to water and texturized dry-calf starter (18 % crude protein, 18.5 % neutral detergent fiber) supplemented with decoquinate at 45 g/ton (Elite 18%, Hubbard feeds, Mankato, Minnesota). Individual starter concentrate intake was recorded weekly and milk intake daily by measuring offers and refusals. Body weight of the calves was measured biweekly 7 h after the morning feeding and following the same order from the beginning of the study until the end at 42 days. Health of calves was monitored daily and those requiring antimicrobial therapy before 42 days were excluded from the study. Fecal samples were collected via rectal palpation from each calf at day 42 and immediately transported to the lab and stored at −20°C. Nasal swabs were also obtained on day 42 by inserting a sterile swab (Puritan HydraFlock, Puritan Diagnostics Llc., Guilford, Maine) into the nasal cavity, always at the left nostril, and rotating 360° several times. After sampling, each nasal swab was transferred into a 1.5 ml plastic vial and frozen at −20°C until nucleic acid extraction.

### DNA Extraction, 16S rRNA Libraries Preparation, and DNA Sequencing

Total nucleic acids were extracted from nasal swabs and 0.3 g of feces mixed with 800 μl and 1,000 μl of LB broth, respectively, using the MagMAX Total Nucleic Acid Isolation Kit (Thermo Fisher Scientific, Grand Island, New York) according to the manufacturer's instructions. The DNA concentration and quality was determined using a Synergy H1/Take 3 spectrophotometer (BioTek, Winooski, Vermont). The DNA quality was assessed by measuring the 260 nm/280 nm and the 260/230 nm ratios of absorbance. A DNA sample was considered pure if the A260/A280 ratio was in the range of 1.8–2 and the A260/A230 ratio was in the range of 2–2.2. Also, the minimum concentration of DNA required for sequencing libraries was 20 ng/μL. All DNA extracts were stored at −20°C and shipped to MR DNA (Shallowater, Texas) for 16S rRNA gene amplification and sequencing on an Illumina MiSeq platform as described below.

The hypervariable regions V1-V3 of the 16S rRNA gene were amplified from each sample by PCR using the universal bacterial primers 27Fmod (5′-AGRGTTTGATCMTGGCTCAG-3′) and 519Rmodbio (5′-GTNTTACNGCGGCKGCTG-3′) with an 8 bp sample-specific barcode on the forward primer. This set of primers produced a fragment approximately 500 bp long. PCR reactions were performed using the HotStarTaq Plus Master Mix Kit (Qiagen, USA) under the following thermocycling conditions: 94°C for 3 min, followed by 28 cycles of 94°C for 30 seconds, 53°C for 40 seconds, 72°C for 1 min, and a final extension at 72°C for 5 min. The resulting PCR products from each sample were visualized by electrophoresis in 2% agarose gels and mixed in equal concentration of DNA for 16S rRNA gene library preparation. The amplicons from pooled samples were then purified using Agencourt AMPure XP beads (Agencourt Bioscience Corporation, Beverly, Massachusetts) and paired-end sequenced (2 × 300) on an Illumina Miseq platform following the manufacturer's instructions.

### Sequencing Data Analysis

The paired-end FASTQ files were joined using a proprietary analysis pipeline by MR DNA (Shallowater, Texas). The joined reads were then processed in QIIME (MacQIIME 1.9.1) (27). All sequence reads were first demultiplexed and quality-filtered with the following parameters: average quality score <25 calculated in sliding window of 25 bp; minimum read length: 450; maximum read length: 550; maximum number of mismatches in primer and barcode sequence: 0; maximum number of ambiguous bases: 0 and maximum homopolymer: 8. The remaining sequences were then clustered into OTUs at 97% similarity using UCLUST and *de novo* and reference-based chimera detection with the intersection method in USEARCH version v5.2.236 (28). For taxonomic assignment, a representative sequence from each OTU was selected and compared with those in the SILVA reference database version 111 (29) using the default taxonomy classifier in QIIME. Singleton OTUs were removed after conducting α-diversity measurements for further analyses. The raw sequencing reads obtained in this study were submitted to the NCBI Sequence Read Archive under the accession number SRP149634.

### Statistical Analysis

To assess the effect of feeding regime on calf bacterial communities both, α and β-diversity parameters were computed. Prior to estimating diversity parameters, all sequence libraries were randomly subsampled separately for each type of sample (fecal and nasal) to the same number of sequences (feces: *n* = 17,327; nasal: *n* = 18,391). To estimate α-diversity parameters, the observed OTUs, Chao1, PD Whole Tree, and Shannon and Goods coverage indexes were calculated for each sample, and rarefaction curves depicted using QIIME (*alpha_diversity.py* and *alpha_rarefaction.py* scripts). For each α-diversity measure and type of sample (fecal and nasal), a non-parametric two sample *t*-test (Monte Carlo with 999 permutations) was performed to assess differences between feeding regimes. For β-diversity analyses, the Unweighted UniFrac distances were calculated for each type of sample (fecal and nasal), and Principal Coordinate Analysis (PCoA) plots performed based on these distances. Relationships between bacterial communities of calves fed either MR or pWM were tested using a one-way analysis of similarity (ANOSIM) with the QIIME python script (*compare_categories.py*). To assess differences in the composition of bacterial communities between feeding regimes, all the OTUs were collapsed into their assigned taxonomy, and then comparisons were done on these taxa assignments. Furthermore, the core microbiome, defined as those OTUs present in all fecal or nasal samples, was determined for both, calves fed MR and pWM, independently one from the other, and its relative abundance compared between feeding regimes. Estimation of *p*-values was performed through the Kruskal–Wallis test using the PROC NPAR1WAY procedure of SAS (version 9.2, SAS Institute Inc., Cary, NC, USA) with a false detection rate correction for multiple hypotheses testing (PROC MULTTEST procedure of SAS) in both bacterial composition analyses.

## Results

Twelve calves fed MR and 9 calves fed pWM required treatment with sulfamethoxazole and trimethoprim for diarrhea, and were therefore excluded from the study. Also, one calf in the pWM treatment group consumed significantly less starter than their counterparts (0.71 vs. 13 kg of concentrate in 42 days of study, respectively), and was therefore removed from the study as well. Therefore, sequencing analysis was performed with 8 calves fed MR and 10 calves fed pWM.

### Calf Performance

Performance data for the 18 healthy calves (8 in MR and 10 in pWM treatment) indicated that calves fed pWM weighed 6.0 kg more (*P* < 0.05) than calves fed MR at 42 days of study (Table 1). From the beginning of the study to day 42, calves fed MR consumed 11 kg of concentrate and calves fed pWM consumed 16 kg, with the gain to feed ratio being greater (*P* < 0.05) in pWM than in MR fed calves (0.77 and 0.71 ± 0.014, respectively) (Table 1).

**Table 1:** Effect of milk feeding regime on growth performance and starter feed intake in dairy calves.

	**Feeding regime**^**1**^			
	**MR (*n* = 8)**	**pWM (*n* = 10)**	**SEM**	***P*-value**
Initial BW, kg	38.8	38.1	1.37	0.723
BW at 42 d, kg	65.8	71.8	1.15	0.002
ADG, kg/d	0.65	0.80	0.028	0.002
Starter DM intake, kg/d	0.27	0.39	0.039	0.047
Total DM intake, kg/d	0.91	1.03	0.047	0.231
Gain to feed ratio	0.71	0.77	0.014	0.011

1 *MR, Milk replacer; pWM, Pasteurized waste milk*.

### Descriptive Data

The total number of 16S rRNA gene sequences generated from both nasal and fecal samples was 2,157,627 with a mean length of 529.6 nucleotides. After removing singletons and poor quality sequences, 1,159,416 reads remained with an average of 32,206 sequences per sample. In total, 10,802 OTUs were identified from fecal samples and 7,878 from nasal swabs by clustering sequences at 97% sequence similarity cut-off. The average number of OTUs per calf was 597 in fecal samples (range 479–777) and, 438 in nasal swabs (range 251–623).

A total of 19 bacterial phyla were identified in the fecal samples. However, the majority of OTUs were assigned to 2 phyla. Overall, 39% of the sequences belonged to the Bacteroidetes phylum, 55.7% to Firmicutes, and 3.3% to Proteobacteria. *Lachnospiraceae* was the most relatively abundant family in the fecal microbiota representing 31.1% of the total sequences, and 58.3% within the Firmicutes phylum. The second and third most relatively abundant families were *Prevotellaceae* (19.6% of the total sequences) with 51.9% of representation in the Bacteroidetes phylum, and *Ruminococcaceae* accounting for 17.8% of the total sequences and 33.2% within the Firmicutes phylum. Within the Proteobateria phylum, *Succinivibrionaceae* was the only family with a relative abundance of more than 1%, representing 2% of the total sequences and 42% of the sequences within the phylum. At the genus level, 124 genera were identified in fecal microbiota. *Prevotella* and *Bacteroides* belong to the Bacteroidetes phylum, and they were the major genera accounting for 15.5% and 9% of all reads, respectively. *Blautia* was the third most abundant genus, and it belongs to the Firmicutes phylum representing 8% of the total reads, and 25.7 % of the *Lachnospiraceae* family.

Regarding the nasal bacterial communities, a total of 18 bacterial phyla were identified. The majority of sequences (97%) were classified into 6 phyla with an average relative abundance of more than 1%. The most abundant phyla were Tenericutes (29.5 %), Firmicutes (19.3%), and Actinobacteria (19%), followed by Proteobacteria, Bacteroidetes, and Fusobacteria (16, 11.5, and 2.5%, respectively). At the family level, only two families had more than 10% relative abundance: *Mycoplasmataceae* (29.5%) and *Microbacteriaceae* (13.7%). Within the Tenericutes phylum, *Mycoplasmataceae* accounted for 99.9% of the sequences, whereas *Microbacteriaceae* represented 72.0% of the Actinobacteria phylum. *Pasteurellaceae* (9.7%) was also a predominant family within the Proteobacteria phylum accounting for 60.6% of the sequences; the other phyla, Bacteroidetes and Firmicutes had more intraspecific diversity being equally represented by at least three different families. At the genus level, 78.3% of the sequences were classified into 259 genera in the nasal microbiota. The most relatively abundant genera were *Pseudoclavibacter* and *Mycoplasma* with 13.8% and 29.5% of the total sequences, respectively. Among the total bacterial genera identified in nasal microbiota, only 11 had a relative abundance of more than 1%.

### Effect of Feeding Regime on α-Diversity Indices

The fecal bacterial communities of calves fed MR did not differ in the number of OTUs, Chao1, PD whole tree, and Shannon diversity index when compared with those from calves fed pWM (Table 2). Similarly, no differences were found for these estimators in the nasal bacterial communities of calves fed either the two feeding regimens (Table 2).

**Table 2:** Alpha-diversity indices (±SE) for both fecal and nasal microbiota communities from calves fed either milk replacer (MR) or pasteurized waste milk (pWM).

	**Fecal microbiota**			**Nasal micrbiota**		
	**MR (*n* = 8)**	**pWM (*n* = 10)**	***P*-value**	**MR (*n* = 8)**	**pWM (*n* = 10)**	***P*-value**
Observed OTUs	623.1 ± 27.38	582.5 ± 18.34	0.18	424.1 ± 41.36	448.5 ± 31.60	0.38
Chao1	802.8 ± 30.50	770.9 ± 24.22	0.20	585.8 ± 48.18	573.2 ± 31.43	0.45
PD whole tree	30.2 ± 0.88	28.7 ± 0.71	0.18	23.6 ± 1.63	25.0 ± 1.23	0.18
Shannon	6.1 ± 0.21	6.4 ± 0.14	0.12	3.4 ± 0.36	3.5 ± 0.46	0.41

### Effect of Feeding Regime on Fecal and Nasal Bacterial Composition

**Figure 1:**
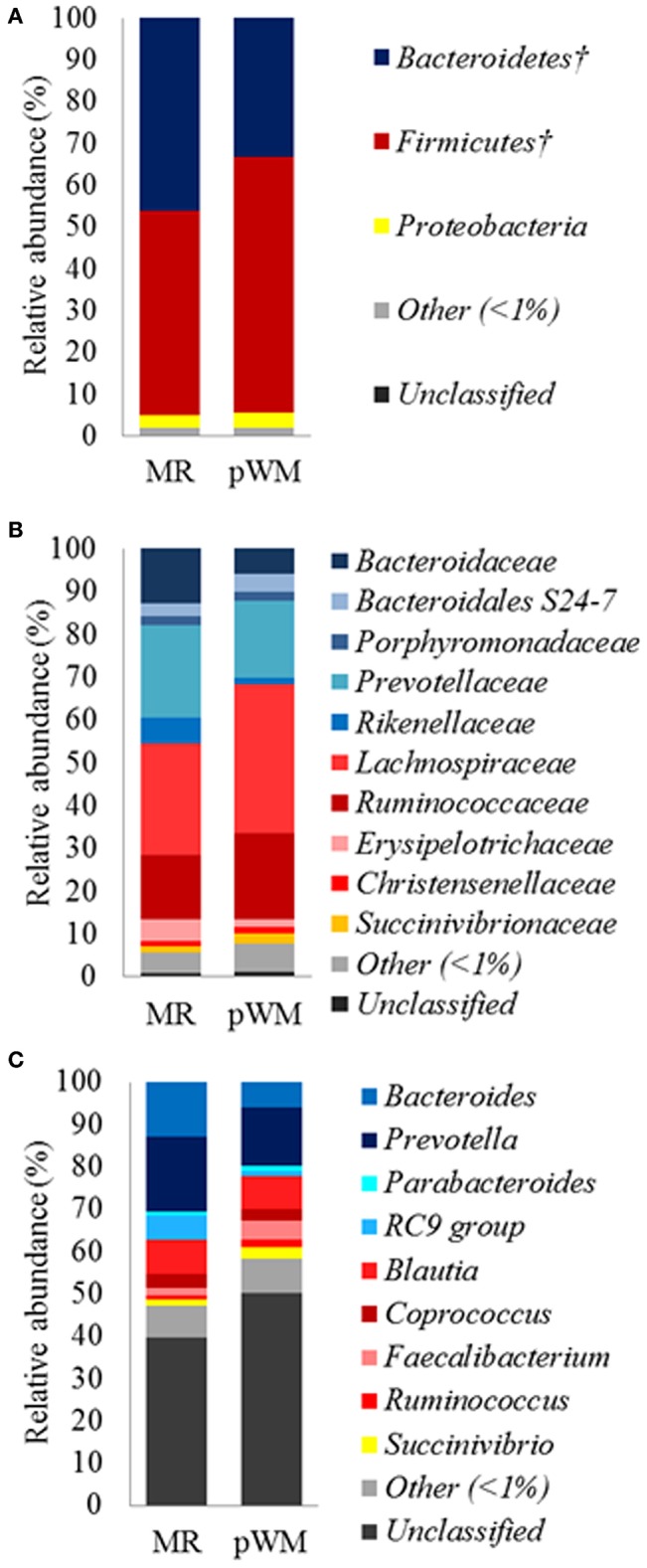
Mean relative abundance (%) of bacterial phyla **(A)**; family **(B)**, and genus **(C)**, in the fecal microbiota of dairy calves fed either milk replacer (MR) (*n* = 8) or pasteurized waste milk (pWM) (*n* = 10). ^†^Indicates that the relative abundances of bacterial divisions tended to be different (*P* < 0.10) between feeding regimes.

The fecal microbiota of calves in the MR treatment tended (*P* = 0.07) to have greater relative abundance of the Bacteroidetes phylum compared with the pWM calves, whereas the relative abundance of Firmicutes tended (*P* = 0.07) to be lower in calves fed MR than in those fed pWM (Figure 1A). No differences in fecal bacterial populations were detected at both, family and genus level between feeding regimes (Figures 1B,C).

**Figure 2:**
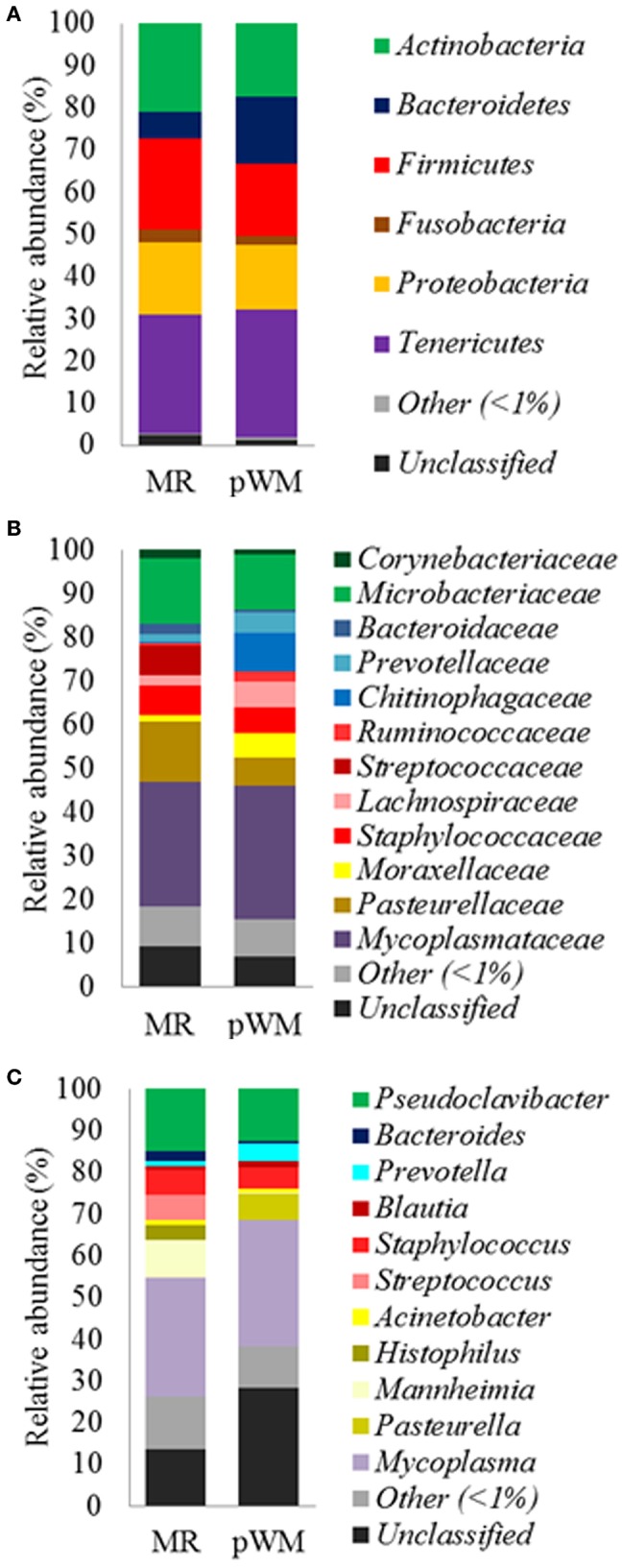
Mean relative abundance (%) of bacterial phyla **(A)**; family **(B)**, and genus **(C)**, in the nasal microbiota of dairy calves fed either milk replacer (MR) (*n* = 8) or pasteurized waste milk (pWM) (*n* = 10).

**Figure 3:**
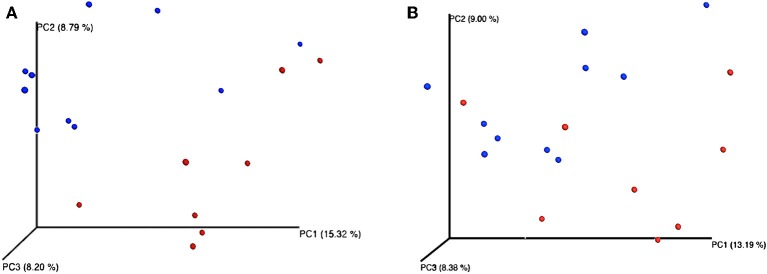
Principal coordinates analysis (PCoA) representing β-diversity of both fecal **(A)** and nasal **(B)** bacterial communities in dairy calves. Figures were computed using unweighted Unifrac distances and a depth coverage of 17,300 sequences per sample. Bacterial communities from calves fed milk replacer (MR) (*n* = 8) are depicted in red, and those from calves fed pasteurized waste milk (pWM) (*n* = 10) in blue.

Feeding regime did not have any effect on the bacterial composition of the nasal microbiota at any of the taxonomic levels tested (phyla, family, and genus) (Figure 2). However, analysis of the unweighted Unifrac distances using PCoA and ANOSIM showed clustering by diet (feces: *R* = 0.331, *P* < 0.05; nasal: *R* = 0.135, *P* < 0.05) (Figure 3).

Although no differences between feeding regimes were observed in the fecal core microbiome, the nasal core microbiome, defined for both MR and pWM fed calves, revealed differences in relative abundances of certain taxa (Table 3). At the family level, the proportion of *Streptococcaceae* was greater (*P* < 0.05) in calves fed MR than in those fed pWM; whereas, at the genus level (*Streptococcus*) this pattern tended (*P* = 0.06) to be different. The *Histophilus* genus was also more relatively abundant (*P* < 0.05) in nasal microbiota of calves fed MR than in those fed pWM. In contrast, the relative abundance of the genus *Prevotella* tended (*P* = 0.07) to be greater in pWM fed calves than in those fed MR.

**Table 3:** The relative abundance of genera in the core nasal microbiota of dairy calves fed either milk replacer (MR) or pasteurized waste milk (pWM).

	**Treatments**			
**Genus**	**MR**	**pWM**	**SEM**	***P*-value**
*Prevotella*	0.19	1.88	0.42	0.06
*Streptococcus*	10.7	0.06	3.09	0.06
*Histophilus*	4.1	0.0	2.05	<0.01

## Discussion

The predominant phyla in feces from preweaned calves are Firmicutes, Bacteroidetes, and Proteobacteria (30). Although in the present study Firmicutes was the most predominant phylum in both feeding treatments, there are inconsistent results in the literature in terms of dominant phyla in preweaned calves. Similar to the present study, Oikonomou et al. (31) found Firmicutes to be the major phylum in feces of preweaning calves, in contrast to Malmuthuge et al. (30) and Deng et al. (15) who reported Bacteroidetes to be the most dominant phylum in samples from the large intestine of calves. However, Edrington et al. (14) reported varying relative abundances of either Firmicutes or Bacteroidetes in preweaning calves at different sampling ages and milk feeding regimes. Furthermore, differential composition between mucosa- and digesta-associated microbiota was also observed throughout the gastrointestinal tract, indicating that the structure of bacterial communities may vary greatly, not only by the region of the gastrointestinal tract sampled, but also depending on the type of sample collected (15, 30). In the present study, differences in the relative abundance of Firmicutes and Bacteroidetes in fecal microbiota might be attributable to three main different aspects between the two treatments: (1) ingredient composition of MR and pWM, and presence of immune factors (i.e., growth factors, cytokines, immunoglobulins) in pWM but not in MR, 2) differences in starter concentrate intake between feeding regimes, and (3) antimicrobial residues present in pWM.

Feeding pWM in the present study improved feed efficiency, probably because of the greater bioavailability of nutrients in whole milk compared with MR (32). A positive relation between an increase in Firmicutes in the gastrointestinal tract and improvements in feed efficiency has been reported in steers and other species (24, 33). Myer et al. (33) found a high relative abundance of Firmicutes within the rumen microbiota of steers with improved feed efficiency, and Looft et al. (24) found a promotion of functional genes related to energy production and conversion in pigs that received medicated feeds, together with a decrease in fecal Bacteroidetes. Similarly, in studies examining the microbiota present in the distal gut of mice, changes in the relative abundance of Bacteroidetes and Firmicutes were found to influence the capacity to harvest energy from the diet (34, 35). Specifically, a greater ratio of Firmicutes to Bacteroidetes was observed in obese mice when compared with their lean counterparts. As calves fed pWM consumed more starter concentrate than calves fed MR, the increase in starter concentrate intake in calves fed pWM together with the greater relative abundance of Firmicutes phylum compared with MR fed calves may be responsible for the tendency toward improved feed efficiency of calves fed pWM.

In infants, breast-feeding is associated with a decrease in microbiota diversity, a decrease in bacteria from the Firmicutes phylum, and some differences in the presence of certain *Bifidobacterium* species compared with formula-fed infants (36). However, Carlisle et al. (37) reported an increase in the relative abundance of Firmicutes in mice fed maternal milk compared with mice fed a milk substitute, as observed herein. Although a decrease in microbiota diversity has been commonly reported in mammals fed milk (37, 38), this effect was not detected in the present study. Generally, pasteurization reduces some of the growth factors, antimicrobial proteins, and immunoglobulins present in raw milk that might contribute to the reduction in microbial diversity observed in breast-fed infants or animals fed maternal milk (39). Thus, one possible explanation for the lack of effect on microbial diversity in pWM calves could be the lower content of these components in pWM. Deng et al. (15) fed calves acidified WM, untreated WM, pWM, and untreated bulk milk to calves, and did not observe differences in microbial diversity in rectal samples. However, a greater relative abundance of beneficial bacteria associated with the production of short-chain fatty acids and involved in important symbiotic host-microbiome relationships were observed in pWM fed calves compared with those fed either untreated or acidified WM. Bach et al. (40) fed raw, pasteurized and UHT milk to calves and did not find differences in total counts of Gram-positive bacteria among diets. However, bacterial counts of *Lactobacillus*, a genus of Gram-positive bacteria that may inhibit the growth of pathogenic bacteria and enhance the immune system response, were found to be lower in both, pasteurized and UHT milk fed calves than in those receiving raw milk. Antimicrobials may have contributed to the change at the phylum level observed herein, but Pereira et al. (41) compared the gut microbiota of calves fed either WM containing a low concentration of a combination of the main antimicrobials found in WM (ampicillin, ceftiofur, penicillin G, and oxytetracycline) or saleable whole milk and did not observe any changes in the microbial profiles at higher taxonomic levels than genus.

The notion that antimicrobials in feed may reduce gut bacterial diversity in livestock has also previously demonstrated in pigs feed a starter concentrate supplemented with antimicrobials (24). However, in the present study, feeding calves pWM containing several classes and variable concentrations of antimicrobial residues (mainly β-lactam antimicrobial residues) did not affect diversity estimators, which were represented by the total number of OTUs, microbial richness (Chao1), diversity (Shannon), and phylogenetic diversity (PD whole tree) of bacterial communities. Similar findings were reported by Pereira et al. (41), who found no differences in microbial diversity between calves fed whole milk with antimicrobials; and those fed antimicrobials-free whole milk, suggesting that the low antimicrobial drug concentration in milk did not exert sufficient pressure to have a significant effect on the gut microbiota.

Although in the current study differences in the composition of the fecal microbiota between feeding regimes were only observed at the phylum level, the PCoA plots and ANOSIM revealed changes in the structure of the bacterial community of calves depending on the type of milk offered (Figure 3). Differences in bacterial communities may not have been observed because the relatively large consumption of solid feed at 42 days of age may has masked the effect of milk-feeding regimes on fecal microbiota at lower taxonomic levels, since feeding starter feed tend to increase the richness of predominant phylotypes along the gastrointestinal tract (42). Similarly, Klein-Jöbstl et al. (43) also reported a high variability in the composition of bacterial communities among calves during the weeks before weaning than either during the first weeks of life or after weaning when gut microbiota became more stabilized. Based on these findings, sampling the calves before the increase in concentrate intake or limiting their concentrate intake before the sampling is concluded would be a better strategy in future studies evaluating the effect of milk feeding regimes on fecal microbiome.

Regarding taxonomic analysis of the nasal microbiota, the most abundant phyla in the respiratory tract of calves was Tenericutes followed by Firmicutes, Actinobacteria, Proteobacteria, and Fusobacteria. Additionally, bacterial communities of the respiratory tract had more intra-individual variability than that found in gut microbiota at each of the taxonomical level assessed. These findings were in agreement with those reported in feedlot calves by Holman et al. (44), who reported a large heterogeneity in the relative abundance of several taxa in the nasopharyngeal tract of cattle. Lima et al. (45), who raised calves under similar environmental conditions compared to those in the present study, detected *Proteobacteria* in young calves (from 3 to 35 days old) as the most abundant bacterial phylum in the upper respiratory tract instead of Tenericutes and Actinobacteria as in the present study.

The present study demonstrated changes in nasal bacterial communities in calves fed two different types of milk. The antimicrobial residues present in pWM might be involved in the changes observed in *Histophilus somni*, the only species in this genus, and *Streptococcus* spp. prevalence. In cattle, antimicrobials such as cephalosporins, tilmicosin, and fluoroquinolones are the first option to treat infectious diseases caused by *H. somni* and *Streptococcus* spp. (46), and most of them were used to treat dairy cows on the study farm where WM was collected. Pereira et al. (41) reported a lower relative abundance of *Streptococcus spp*. and *Clostridium spp*. in the fecal microbiota of calves fed whole milk containing sub-minimum inhibitory concentrations (MIC) of ceftiofur, penicillin, ampicillin, and oxytetracycline, suggesting these genera are highly sensitive to sub-MIC of antimicrobials. Differences in relative abundance of *Prevotella* herein are difficult to understand. In pigs, the presence of *Prevotella* in the respiratory tract has been associated with farms without respiratory disease (47).

Our results show that feeding pWM in commercial conditions with several classes and variable concentrations of antimicrobial residues affects the composition of the fecal and nasal microbiota of pre-weaned calves, with these effects being more evident in the respiratory tract. Furthermore, feeding pWM to calves instead of antimicrobial-free MR with a similar nutrient composition to WM, improves feed efficiency and starter concentrate intake inducing changes in the relative abundance of the Firmicutes and Bacteroidetes phyla in the gut microbiota. However, these findings have to be interpreted cautiously since nutrient quality and the amount and type of antimicrobials residues present in WM may vary greatly among dairy farms and over time.

## Data Availability

The datasets generated for this study can be found in NCBI Sequence Read Archive, the accession number SRP149634.

## Ethics Statement

The current study was conducted at the Southern Research and Outreach Center (SROC) of the University of Minnesota from July to November 2014 and it was approved by the Animal Care Committee under the protocol number 1407-31648A.

## Author Contributions

GM conducted sampling procedure and laboratory analysis. GM and MT conducted data analysis and wrote the manuscript. MT, HC-J, and AB reviewed critically the manuscript for final approval of the version to be published.

### Conflict of Interest Statement

The authors declare that the research was conducted in the absence of any commercial or financial relationships that could be construed as a potential conflict of interest.
